# Fucoxanthin Inhibits Myofibroblast Differentiation and Extracellular Matrix Production in Nasal Polyp-Derived Fibroblasts via Modulation of Smad-Dependent and Smad-Independent Signaling Pathways

**DOI:** 10.3390/md16090323

**Published:** 2018-09-10

**Authors:** Hyun Jung, Dae-Sung Lee, Seong Kook Park, Jung Sik Choi, Won-Kyo Jung, Won Sun Park, Il-Whan Choi

**Affiliations:** 1 Department of Otorhinolaryngology-Head & Neck Surgery, Inje University College of Medicine, Busan 47392, Korea; jh9002@hotmail.com (H.J.); sinus4@paik.ac.kr (S.K.P.); 2 Department of Applied Research, National Marine Biodiversity Institute of Korea, Seocheon 33662, Korea; daesung@mabik.re.kr; 3 Department of Internal Medicine, Busan Paik Hospital, Inje University, Busan 47392, Korea; cwj1225@naver.com; 4 Department of Biomedical Engineering, Center for Marine-Integrated Biomedical Technology (BK21 Plus) Pukyong National University, Busan 48513, Korea; wkjung@pknu.ac.kr; 5 Department of Physiology, Kangwon National University School of Medicine, Chuncheon 24341, Korea; 6 Department of Microbiology and Immunology, College of Medicine Inje University, Busan 47392, Korea

**Keywords:** nasal polyps, fucoxantin, transforming growth factor, extracellular matrix accumulation, fibroblasts

## Abstract

Nasal polyps (NPs) are a multifactorial disorder associated with a chronic inflammatory state of the nasal mucosa. Fucoxanthin (Fx) is a characteristic orange carotenoid obtained from brown algae and has diverse immunological properties. The present study investigated whether Fx inhibits fibrosis-related effects in nasal polyp-derived fibroblasts (NPDFs) and elucidated the molecular signaling pathways involved. The production of collagen type I (Col-1) was investigated in NP tissue via immunohistochemistry and western blot analysis. NPDFs were treated with transforming growth factor (TGF)-β1 (1 ng/mL) in the presence or absence of Fx (5–30 µM). The levels of α-smooth muscle actin (α-SMA), Col-1, and phosphorylated (p)-Smad 2/3, signal protein-1 (SP-1), MAPKs (mitogen-activated protein kinases), and Akt were measured by western blot analysis. The expression of Col-1 was detected in NP tissues. TGF-β1 stimulated the production of α-SMA and Col-1, and stimulated the contraction of collagen gel. However, pretreatment with Fx attenuated these effects. Furthermore, these inhibitory effects were mediated through modulation of both Smad 2/3 and Akt/SP-1 signaling pathways in TGF-β1-induced NPDFs. The results from the present study suggest that Fx may be a novel anti-fibrotic agent for the treatment of NP formation.

## 1. Introduction

The nose is the first point of contact of environmental microbes, pollutants, and allergens before reaching the respiratory system; therefore, it is not surprising that inflammation in the upper airway is a common disorder. Nasal polyps (NPs) are a multifactorial disorder associated with chronic inflammation of the nasal mucosa. NPs cause morbidity including rhinorrhea, loss of smell, and headache [1,2]. The typical histomorphological features of NPs are nonspecific soft tissue edema with hyperplasia of goblet cells, formation of atypical glands, thickening of the basement membrane, and infiltration of various inflammatory cells [3,4,5]. Most physicians suggest either the use of a long-term intranasal corticosteroid in conjunction with a short course of oral steroids, or surgery in many cases [6]. Despite successful surgery, there is a high possibility of disease recurrence which may require reoperation. Despite considerable efforts, the etiology and pathophysiology of NPs has never been addressed in detail. The process of NP formation is proposed to commence with injury to the mucosal epithelium, followed by accumulation of extracellular matrix (ECM) proteins and matrix metalloproteinases (MMPs), and finally, infiltration of various inflammatory cells [7]. These cells produce both fibers and amorphous ground substance.

Fibroblasts are a common cell type in connective tissues and are found in every tissue of the body. They produce and respond to various inflammatory cytokines [8]. They are the central mediators of ECM accumulation, and cell differentiation and proliferation that occur in response to prolonged tissue damage [9]. Such tissue damage often stimulates differentiation of fibroblasts to myofibroblasts which participate in the inflammatory response to injury [10]. Differentiation of fibroblasts into myofibroblasts defines a physiological process that facilitates the formation of NPs [11]. Myofibroblasts are generally identified by their expression of α-smooth muscle actin (α-SMA). A previous study has suggested that damage to the mucosal epithelium induces the expression of transforming growth factor (TGF)-β1 [12]. TGF-β1 participates in fibrosis in conditions such as diabetic nephropathy, Crohn’s disease, myocarditis, and rheumatoid arthritis [13]. The stimulation of TGF-β1 is characterized by an increase in the accumulation of ECM components as well as motility and invasion, migration and accumulation, angiogenesis, and an immune response [13,14]. Additionally, TGF-β1 is an important profibrotic cytokine closely related to the activation and differentiation of fibroblasts. It has been shown that NPs express high levels of TGF-β1 [15]. In addition, excess deposition of ECM is found in NPs [16]. 

**Figure 1:**
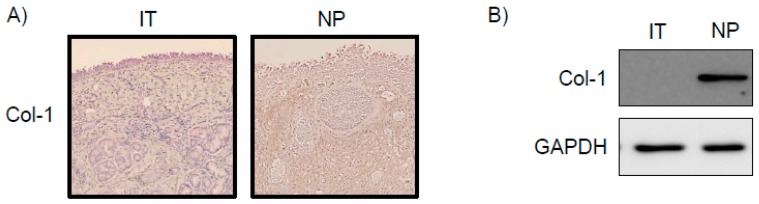
Expression of collagen type I (Col-1) in nasal polyp (NP) tissues. (**A**) The distribution of Col-1 in the samples of NP tissues as examined via immunohistochemical staining using anti-Col-1 antibody; (**B**) Western blot analysis of NPs. GAPDH was used as an internal control. IT, inferior turbinate tissue; NP, nasal polyp tissue; GAPDH, Glyceraldehyde 3-phosphate dehydrogenase.

Marine algae are a source of bioactive compounds with both medicinal and nutritional value and have traditionally been used in Asian countries for a long time. Marine algae are classified into three classes: green, red, and brown. Among these, brown algae contain many types of physiologically active compounds such as omega-3 polyunsaturated fatty acids (PUFAs), arachidonic acid, polyphenols, polysaccharides, and fucoxanthin (Fx) [17]. Fx (Figure 1) is a characteristic carotenoid obtained from brown algae [18]. Fx exhibits anti-inflammatory, radical scavenging, anti-mutagenic, anti-diabetic, anti-obesity, and anti-cancer properties [18]. However, to date, the anti-fibrotic effect of Fx in NP formation has not been reported. Based on the above studies, we investigated the possibility of Fx inhibiting myofibroblast differentiation and ECM accumulation in TGF-β1-stimulated nasal polyp-derived fibroblasts (NPDFs).

## 2. Results

### 2.1. Expression of Col-1 in NP Tissues

To explore whether Col-1 was expressed in NP tissues, immunohistochemistry was performed in inferior turbinate (IT) tissues and NP tissues. In NP tissues, Col-1 immunoreactivity was detected in lesions in which overall stroma was observed (Figure 1A). Additionally, western blotting revealed a high expression of Col-1 proteins in NP tissue lysates (Figure 1B). However, IT tissues did not express Col-1 proteins.

### 2.2. Effects of Fx on the Viability of NPDFs

**Figure 2:**
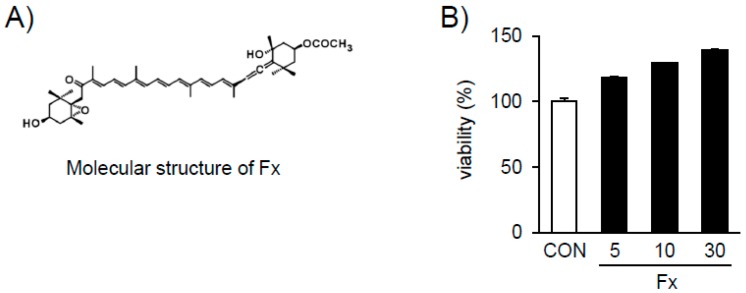
Chemical structure of fucoxanthin (Fx) and the effect of Fx on nasal polyp-derived fibroblast (NPDF) viability. (**A**) Chemical structure of Fx. (**B**) Treatment of cells with various concentrations (5–30 µM) of Fx for 30 min. Cell viability was assessed using the Cell Counting Kit-8 (CCK-8) assay. The results are expressed as the percentage of surviving cells relative to the untreated cells (CON). Each value indicates the mean ± S.E.M. and is representative of results obtained from three independent experiments.

The viability of NPDFs treated with Fx (Figure 2A) was conducted using the Cell Counting Kit-8 (CCK-8) assay. The CCK assay revealed that Fx is non-toxic to NPDF under 30 μM (Figure 2B).

### 2.3. Effects of Fx on the Production of α-SMA and Col-1 in TGF-β1-Stimulated NPDFs

**Figure 3:**
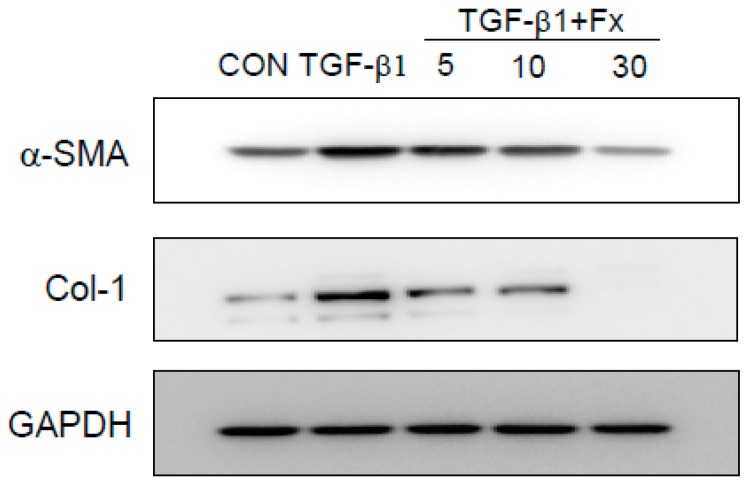
Effects of fucoxanthin (Fx) on α-smooth muscle actin (α-SMA) and Col-1 protein expression in transforming growth factor (TGF)-β1-stimulated NPDFs. The cells were seeded at 2 × 10^5^ cells/mL and incubated with various concentrations (5, 10, and 30 µM) of Fx for 1 h, followed by TGF-β1 stimulation (1 ng/mL). After stimulation with TGF-β1 for 24 h, α-SMA and Col-1 protein expression was determined via western blotting. Untreated cells were used as a control (CON) and GAPDH was used as an internal control. Each value indicates the mean ± S.E.M. and is representative of results obtained from three independent experiments.

We investigated whether Fx attenuates the TGF-β1-induced expression of α-SMA and Col-1 proteins in NPDFs. Production of α-SMA and Col-1 proteins was significantly inhibited in an Fx dose-dependent manner (Figure 3).

### 2.4. Fx Inhibits TGF-β1-Stimulated Smad-2/3-Dependent and Smad-2/3-Independent Signaling Pathways

**Figure 4:**
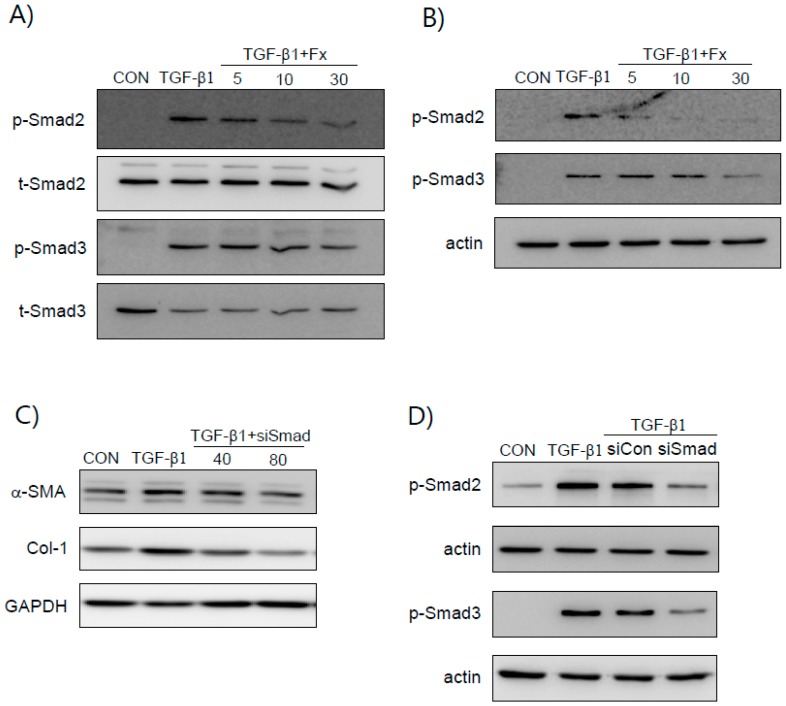
Effects of fucoxanthin (Fx) on Smad2/Smad3 activation in NPDFs. (**A**) The NPDFs were pretreated with Fx (5–30 µM) for 30 min, followed by stimulation with TGF-β1 (1 ng/mL) for 30 min. (**B**) The Smad 2/3 nuclear translocation was determined via western blot analysis. (**C**) Smad 2/3 silencing inhibits the expression of α-SMA and Col-1 proteins in TGF-β1-stimulated NPDFs. The NPDFs were transfected with Smad2/3 siRNA (40 and 80 nM) for 24 h, followed by stimulation with TGF-β1 (1 ng/mL) for 24 h. (**D**) The NPDFs were transfected with either the control siRNA (80 nM) or the Smad 2/3 siRNA (80 nM) for 24 h, followed by stimulation with TGF-β1 (1 ng/mL) for 30 min. Each bar represents the mean ± S.E.M. from three independent experiments. Untreated cells were used as a control (CON). p-Smad 2/3, phosphor-Smad 2/3; t-Smad 2/3, total-Smad 2/3; siCon, control siRNA; siSmad, Smad 2/3 siRNA.

**Figure 5:**
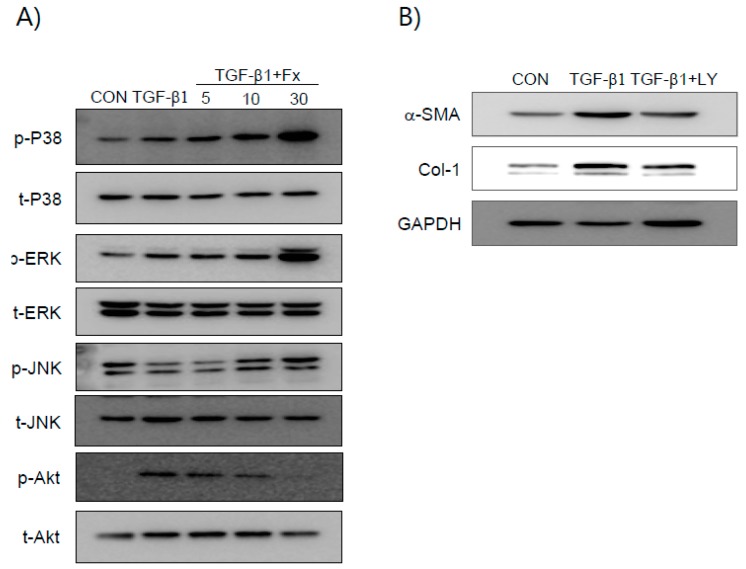
Effects of fucoxanthin (Fx) on TGF-β1-induced phosphorylation of ERK-1/2, SAPK/JNK, p38 MAP kinase, and Akt in NPDFs. The NPDFs were treated with the indicated concentrations of Fx for 2 h, followed by stimulation with TGF-β1 (1 ng/mL) for 30 min. (**A**) Cell extracts were prepared and subjected to western blotting with antibodies specific for the phosphorylated forms of ERK, JNK, p38, and Akt. The results are representative of three independent experiments. (**B**) After treatment with TGF-β1 for 24 h in the presence or absence of LY294002 (LY) (20 μM), the cell extracts were isolated and western blot analysis of α-SMA and Col-1 proteins was performed. Untreated cells were used as a control (CON). ERK-1/2, extracellilar signal-regulated kinase-1/2; SAPK/JNK, Stress-activated protein kinase/c-Jun NH2-terminal kinase; p38 MAP kinase, p38 mitogen-activated protein kinase.

We examined whether Fx inhibits the TGF-β1 signaling pathway. Smad 2 and Smad 3 phosphorylation in NPDFs was increased by TGF-β1 induction (Figure 4), whereas when the NPDFs were pretreated with Fx, this phosphorylation was reduced. TGF-β1 is also known to induce non-canonical signal responses, such as MAPKs- and phosphoinositide 3-kinase/Protein kinase B (PI3K/Akt)-mediated signaling pathways [19]. Fx suppressed phosphorylation of PI3K/Akt (Figure 5A). However, Fx did not show any inhibitory effect on the phosphorylation of MAPKs. The TGF-β1-induced production of α-SMA and Col-1 was significantly attenuated by LY294002 (a PI3K/Akt inhibitor) (Figure 5B).

### 2.5. Fx Inhibits TGF-β1-Induced Signal Protein-1 (SP-1) Activation

**Figure 6:**
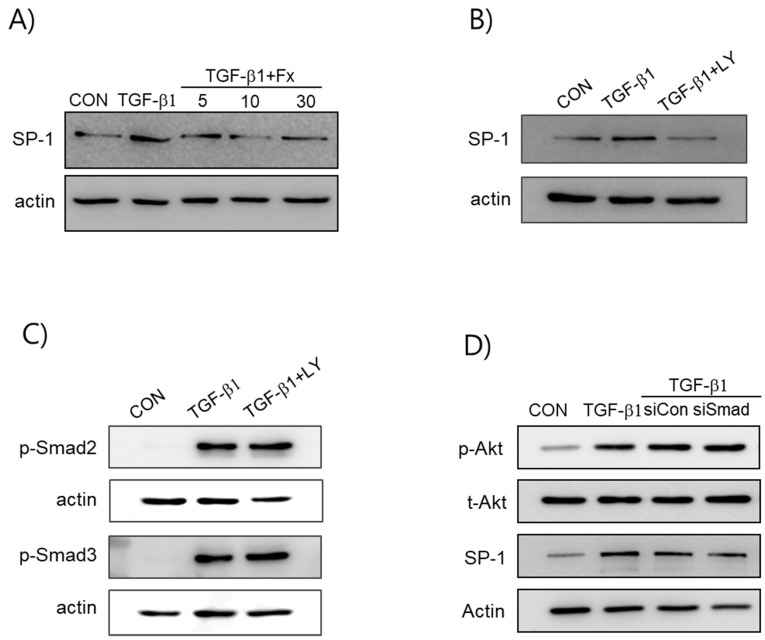
Effects of fucoxanthin (Fx) on signal protein-1 (SP-1) activity in TGF-β1-induced NPDFs. (**A**) Cells were pretreated with Fx (5–30 µM) for 1 h before stimulation with TGF-β1 (1 ng/mL) for 30 min. Nuclear extracts were prepared and analyzed for SP-1 activity via western blotting. (**B**) Cells were incubated with 20 μM LY294002 for 1 h followed by TGF-β1 treatment for 30 min, and nuclear extracts were prepared and analyzed for SP-1 activity via western blotting. (**C**) After pretreatment with 20 μM LY294002 for 1 h, the nuclear proteins were isolated after a 30 min interval and Smad 2 and Smad 3 nuclear translocation was determined via western blot analysis using antibodies specific to the phosphorylated forms of Smad 2 and 3. (**D**) The NPDFs were transfected with either the control siRNA (80 nM) or the Smad 2/3 siRNA (80 nM) for 24 h, followed by stimulation with TGF-β1 (1 ng/mL) for 30 min. Untreated cells were used as a control (CON).

The expression of Col-1 is induced by the binding of signal protein-1 (SP-1) to the Col-1 gene. Thus, to elucidate the mechanisms of Fx affecting the expression of fibrotic factors, we examined the effects of Fx on the activation of SP-1. Induction with TGF-β1 led to a significant increase in the translocation of SP-1 into the nuclei, whereas pretreatment with Fx markedly reduced the TGF-β1-stimulated nuclear translocation of SP-1. Subsequently, to elucidate the upstream pathways of SP-1, we treated the cells with LY294002. We found that the treatment with LY294002 significantly inhibited the translocation of SP-1 into the nuclei. Furthermore, we explored whether the PI3K/Akt signaling pathway is involved in the Smad pathways. As presented in Figure 6C, LY294002 did not inhibit the translocation of Smad 2/3 into the nuclei. Also, the siRNA-mediated silencing of Smad2/3 did not inhibit TGF-β1-induced Akt phosphorylation and SP-1 activation (Figure 6D). Therefore, the modulation effects of Fx on fibrotic markers occur via at least two different signaling pathways.

### 2.6. Synergistic Effects of Smad and SP-1 Signal Pathways on the Production of α-SMA and Col-1 Proteins

**Figure 7:**
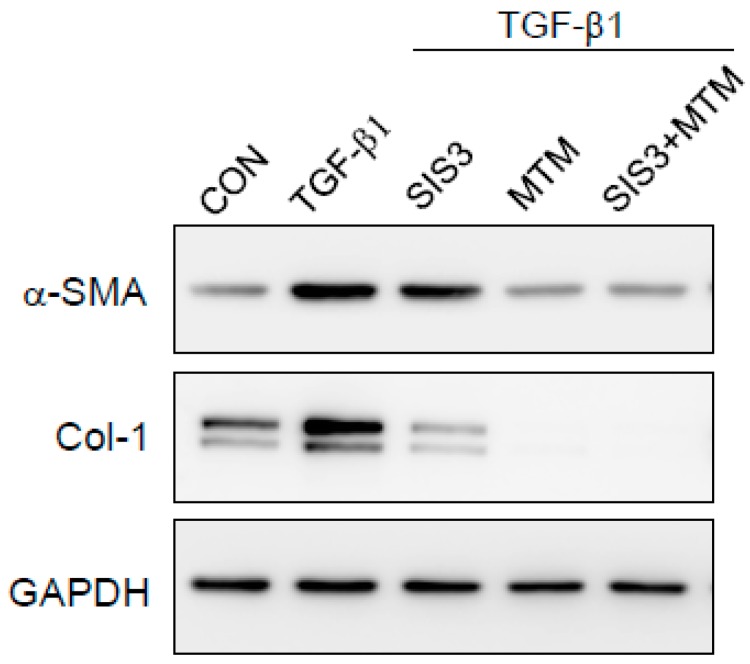
Synergistic effect of Smad and SP-1 on the expression of α-SMA and Col-1 proteins in TGF-β1-stimulated NPDFs. The cells were seeded at 2 × 10^5^ cells/mL and incubated with SIS3 (Smad 3 inhibitor), mithramycin (MTM) (SP-1 inhibitor), and SIS3 + MTM for 1 h prior to TGF-β1 stimulation (1 ng/mL). Following stimulation with TGF-β1 for 24 h, α-SMA protein and Col-1 protein expression was determined via western blotting. Untreated cells were used as a control (CON) and GAPDH was used as an internal control. Each value indicates the mean ± S.E.M. and is representative of results obtained from three independent experiments.

We hypothesized that the inhibition of both Smad and SP-1 signal pathways can produce a synergistic effect. As shown in Figure 7, inhibition of both Smad and Sp-1 using the indicated inhibitors [SIS3 (Smad 3 inhibitor) and mithramycin, respectively] more effectively reduced the expression of Col-1 and α-SMA proteins than inhibition of Smad or Sp-1 alone.

### 2.7. Effects of Fx on the Fibroblast Contractile Activity

**Figure 8:**
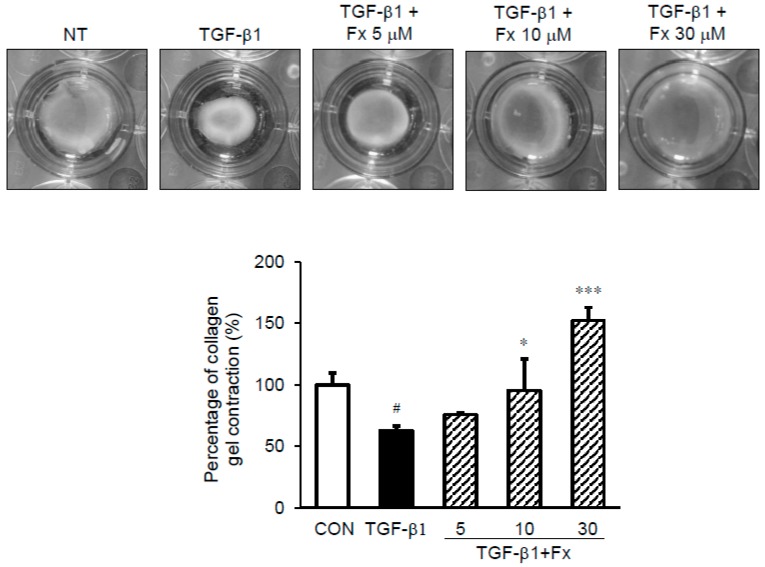
Effect of fucoxanthin (Fx) on the contractile activity of NPDFs. TGF-β1 induction decreased the size of the gel, while Fx pretreatment inhibited this reduction. Data is presented in the form of the percentage of gel surface area in each well to the control gel surface area. ^#^ *p* < 0.05 vs. control group (CON, no treatment); * *p* < 0.05 and *** *p* < 0.001 vs. TGF-β1-stimulated group.

A collagen type I gel contraction assay was performed to evaluate whether Fx blocks the TGF-β1- induced contraction activity of NPDFs. While stimulation with TGF-β1 reduced the size of the collagen gel (63.16% vs. TGF-β1-untreated group (100%)), pretreatment with Fx blocked this effect (Fx: 75.83%, 95.42%, 152.10% for 5, 10, and 30 μM, respectively vs. TGF-β1-untreated group) (Figure 8).

## 3. Discussion

Emerging evidence indicates that various natural compounds have protective effects against inflammatory responses. NPs are thought to occur as a result of inflammation of the lining of the nasal cavity and sinus. On the basis of the proposed anti-inflammatory effects of Fx, we attempted to determine whether Fx offers protection from myofibroblast differentiation and collagen type I expression using TGF-β1-induced NPDFs. Additionally, we explored the mechanisms involved in the inhibitory effects of Fx, such as fibrosis-associated pathways.

It is believed that fibroblast effector functions and phenotypic changes play an important role in the remodeling process. A fibroblast phenotype associated with chronically inflamed tissue is known as myofibroblast [9]. It has been proven that fibroblasts and myofibroblasts play an important role in the remodeling process associated with chronic rhinosinusitis with NPs (CRSwNPs) [16]. Myofibroblasts are considered to play a critical role in excessive matrix deposition during the fibrotic process [20]. The possible contribution of myofibroblasts to the disordered extracellular matrix production associated with asthma and pulmonary fibrosis suggests that they could also be involved in NP where a similar structural abnormality has been observed [3,6]. The presence of myofibroblasts is well established in NPs, whereas they are nearly absent from normal turbinate tissue. Therefore, myofibroblasts play a crucial role in the pathogenesis of NPs.

Myofibroblasts express α-SMA, Col-1, and fibronectin, and proliferate and show contractile properties [21]. It is well known that ECM accumulation may be a crucial factor in the pathogenesis of NP formation [22]. α-SMA expression is the defining characteristic of mature myofibroblasts, and has been shown to increase fibroblast contractile activity and to decrease fibroblast motility. Collagen is the major structural protein in the extracellular space of various connective tissues in animals [23]. Additionally, collagen is involved in the processes of growth, differentiation, and wound healing. Elongated fibrous collagen is found mostly in fibrous tissues such as tendons, ligaments, and skin [23]. Additionally, the deposition of Col-1 was increased in NPs compared with normal control nasal turbinate tissue. Therefore, we explored the inhibitory effects of Fx on myofibroblast differentiation, Col-1 expression, and collagen contraction in TGF-β1-stimulated NPDFs at non-cytotoxic concentrations.

It has been reported that damage to the mucosal epithelium induces the expression of TGF-β1 [6]. TGF-β1, which is expressed in high levels in NP tissues, is related to structural modifications that characterize NP formation [24]. In previous studies, TGF-β1 increased the protein levels of α-SMA and Col-1 in NPDFs [1,25]. As expected, the expression of α-SMA and Col-1 in NPDFs was significantly increased by TGF-β1 induction in our experimental system. However, the increased levels of α-SMA and Col-1 were significantly suppressed in a dose-dependent manner by treatment with 5–30 μM Fx without cytotoxicity (Figure 3). This result suggests that Fx attenuates the TGF-β1-stimulated production of α-SMA and Col-1 by suppressing the TGF-β1-associated signaling pathways. It is well known that TGF-β1 activates pro-fibrotic responses mainly through the Smad signaling pathways. Smads are canonical components in the signaling pathways of TGF-β family members. Therefore, regulation of the Smad pathways provides an effective therapeutic strategy for NPs. Upon stimulation by TGF-β1, Smads are phosphorylated by specific cell surface receptors (type I and type II receptors) that interact with the TGF-β receptor complex, causing Smad 2/3 to oligomerize with Smad 4 and translocate to the nucleus where they activate the transcription of TGF-β responsive genes [26]. In this regard, we investigated whether Fx inhibits the phosphorylation of Smad 2/3 in the cytosol. Next, we investigated whether Fx attenuates the translocation of Smad 2/3 to the nucleus. As shown in Figure 4A,B, treatment with Fx ameliorated this phosphorylation and translocation of Smad 2/3 in a dose-dependent manner, respectively. To verify whether TGF-β1 induces the expression of α-SMA and Col-1 through the Smad 2/3 signaling pathway, we used an siRNA against Smad 2/3 under TGF-β1 stimulation. As expected, our results showed that the siRNA against Smad 2/3 inhibited the expression of α-SMA and Col-1 in TGF-β1-stimulated NPDFs, suggesting that Fx may be useful for α-SMA and Col-1 production in NPs through the modulation of Smad 2/3 signal activation (Figure 4). These results are in line with a previous study that reported that TGF-β1 induced production of α-SMA and Col-1 involved in the activation of the Smad 2/3 pathways in NPDFs [27].

It is well known that the TGF-β mechanism proceeds through both Smad-dependent and Smad-independent pathways [28]. In general, Smad-dependent signaling is required for a complete response to TGF-β, but it is not enough. Therefore, we analyzed the participants of non-Smad pathways in TGF-β1-mediated signaling, especially that of the PI3K/Akt- and MAP kinase pathways, underlying the anti-fibrotic effects of Fx. We observed that Fx attenuates PI3K/Akt activation, whereas pretreatment with Fx did not downregulate TGF-β1-stimulated MAPK phosphorylation (Figure 5). As expected, the production of α-SMA and Col-1 was attenuated by treatment with a PI3K inhibitor (LY294002) in response to TGF-β1 stimulation in the NPDFs. Further, we investigated the downstream pathway of PI3K/Akt signaling. Signal protein-1 (SP-1), which is a ubiquitously expressed transcription factor, regulates multiple biological processes. Additionally, SP-1 is a transactivator of both TGF-β receptors and PI3K/Akt signaling pathways [29]. Several studies have reported the critical role of SP-1 in inducing the deposition of Col-1 during the progression of fibrosis [30,31]. In line with previous reports, we found that SP-1 was attenuated by Fx treatment in TGF-β1-induced NPDFs. We also observed that SP-1 was modulated by LY294002 treatment. Furthermore, we have investigated whether or not the PI3K/Akt signaling pathway is associated with the Smad signaling pathway. In this study, the inhibition of the PI3K/Akt pathway using LY294002 did not appear to affect Smad2/3 translocation in NPDFs. Therefore, we have found that the PI3K/Akt signaling pathway is not associated with the Smad-dependent signaling pathway (Figure 6C). Next, we studied whether these two signaling mechanisms (Smad-dependent and Smad-independent) have synergistic cooperative effects. As shown in Figure 7, the inhibition of α-SMA and Col-1 was more efficiently achieved by a combination of both inhibitors (Smad 3 (SIS3) and SP-1 (MTM) inhibitors) than by treatment with either inhibitor alone. This result suggests that the suppressive effects of Fx on the production of α-SMA and Col-1 are synergistic through combined inhibition of the Smad-dependent and Smad-independent (PI3K/Akt) signaling pathways.

As previously suggested in this study, α-SMA is considered a credible marker of myofibroblastic differentiation [32]. This suggests that there is a relationship between the production of α-SMA and the enhanced contractile capacity of myofibroblasts. Therefore, we examined the effect of Fx on collagen gel contraction. Currently, cultures of fibroblasts in a three-dimensional collagen gel have been used as tissue contraction models that characterize fibrosis, such as under in vivo conditions [32]. We successfully constructed the nasal polyp fibrosis model using TGF-β1 stimulation in vitro. As shown in Figure 8, the contractile capacity of fibroblasts pretreated with Fx was increased compared to that of normal fibroblasts treated with only TGF-β1; this was apparent after observing the attenuation in the gel contraction after treatment with Fx. This study demonstrated that Fx has an inhibitory effect on myofibroblast differentiation and ECM expression in TGF-β1-stimulated NPDFs.

In conclusion, we demonstrated that Fx effectively suppresses the production of profibrotic markers such as α-SMA and Col-1, and collagen gel contraction in vitro using TGF-β1-induced NPDFs. In addition, these inhibitory activities of Fx were mediated through the modulation of both Smad 2/3 and PI3K/Akt/SP-1 signaling pathways. These results imply that Fx may be a suitable compound for the treatment of NP formation.

## 4. Materials and Methods

### 4.1. Reagents

We obtained Fx, mithramycin A, and SIS3 from Sigma-Aldrich (St. Louis, MO, USA). TGF-β1 was obtained from R&D Systems (Minneapolis, MN, USA). Cell Counting Kit-8 was obtained from Dojindo Laboratories (CCK-8, Kumamoto, Japan). Antibodies against α-SMA (cat. no. ab5694) and Col-1 (cat. no. ab88147) were obtained from Abcam Inc. (Cambridge, MA, USA). Antibody against actin (cat. no. 612656) was obtained from BD Biosciences (San Jose, CA, USA). Antibody against GAPDH (cat. no. LF-PA0018) was purchased from Young In Frontier (Seoul, Korea). Antibody against SP-1 (cat. no. sc-420) was purchased from Santa Cruz Biotechnology Inc. (Santa Cruz, CA, USA). Antibodies against Smad 2 (cat. no. 5339), p-Smad 2 (cat. no. 3101), Smad 3 (cat. no. 9523), and p-Smad 3 (cat. no. 9520) were purchased from Cell Signaling Technology, Inc. (Danvers, MA, USA). We purchased Smad 2/3-specific small interfering RNAs (siRNAs, cat. no. sc-37238) and control siRNA (cat. no. sc-37007) from Santa Cruz Biotechnology Inc. (Santa Cruz, CA, USA). Rat tail type I collagen was purchased from BD Biosciences (San Jose, CA, USA).

### 4.2. NP-Derived Fibroblast Culture

Patients with NPs were recruited and NPDFs were isolated and cultured. The study was approved by the Local Ethics Committee of Inje University, Busan Paik Hospital, Busan, Republic of Korea. NPDFs were isolated from surgical tissues by enzymatic digestion with collagenase (500 U/mL; Sigma-Aldrich; Merck KGaA, Darmstadt, Germany), hyaluronidase (30 U/mL; Sigma-Aldrich; Merck KGaA, Dermstadt, Germany), and DNase (10 U/mL; Sigma-Aldrich; Merck KGaA, Dermstadt, Germany). Cells were cultured in Dulbecco’s Modified Eagle’s medium (DMEM) containing 10% (*v*/*v*) heat-inactivated fetal bovine serum (Invitrogen; Thermo Fisher Scientific, Inc., Waltham, MA, USA), 1000 U/mL penicillin, and 1000 µg/mL streptomycin (Invitrogen; Thermo Fisher Scientific, Inc., Waltham, MA, USA) at 37 °C in an atmosphere containing 5% CO_2_. The purity of the NPDFs was confirmed via flow cytometry and the characteristic spindle-shaped cell morphology of the cells. Cells were used in the fourth to sixth cell passage.

### 4.3. Immunohistochemistry

Individuals were diagnosed with NPs based on the minimal criteria for chronic rhinosinusitis with NPs. A total of six subjects (male:female, 5:1; median age, 43) with NPs and six subjects with deviated nasal septa were recruited from the Department of Otorhinolaryngology, Inje University Pusan Paik Hospital (Pusan, Korea). Written informed consent was obtained from each patient and the study was approved by the ethics committees of Inje University Pusan Paik Hospital. A NP was defined as the presence of endoscopically visible bilateral polyps growing from the middle nasal meatus into the nasal cavities, and affecting the ethmoid and maxillary sinuses on computed tomography (cT) of the paranasal sinus. NPs were obtained from the region of the middle meatus at the beginning of the surgical procedure. As a control, nasal mucosal tissue was also obtained from the inferior turbinate (IT) of patients who underwent a septoturbinoplasty. The subjects had no history of nasal allergy, asthma, or aspirin sensitivity. The diagnosis of allergy was based on both a history of allergy and the results of comparison of serum-specific IgE (ImmunocAP) or skin prick tests. No patient had received steroids (systemic or topical), nonsteroidal anti-inflammatory drugs, anti-histamines, or macrolide antibiotics during the four weeks before the biopsy. NP sections (5 μm thick) were prepared from formalin-fixed paraffin-embedded tissue. The sections were incubated overnight with Col-1 antibody at 4°C. The slides were then incubated with anti-mouse IgG-HRP for 1 h at room temperature in the dark. 3,3′-Diaminobenzidine (DAB) was used as a chromogen, and Mayer’s hematoxylin was used for counterstaining. The expression levels of Col-1 were evaluated under a digital slide scanner (NanoZoomer 2.0-RS; Hamamatsu, Shizuoka, Japan).

### 4.4. Determining Cell Viability

The cell viability of Fx was evaluated using the CCK-8 method. Briefly, 96-well microplates containing NPDFs (8 × 10^4^ cells/well) were cultivated in DMEM. The cells were incubated with various concentrations of Fx for 24 h at 37 °C. Next, the cells were incubated with CCK-8 for 1 h. The absorbance was recorded at 450 nm using a microplate reader (SpectraMax M2e, Molecular Devices, Sunnyvale, CA, USA).

### 4.5. Western Blot Analysis

Equal quantities of protein were separated on 10% sodium dodecyl sulfate (SDS)-polyacrylamide mini-gels and transferred to nitrocellulose membranes. After overnight incubation with the specific primary antibodies, the membranes were incubated with a horseradish peroxidase-conjugated secondary antibody at room temperature for 1 h. The staining intensity of the bands was visualized using an enhanced chemiluminescence (ECL) detection system (Pierce Biotechnology, Inc., Rockford, IL, USA) and band intensities were evaluated quantitatively using the Multi Gauge version 2.2 software (Fuji Film, Tokyo, Japan).

### 4.6. Silencing of Smad 2/3 by Synthetic siRNAs

Sixteen hours after plating, the cells were transfected with either Smad 2/3-siRNAs or control siRNA using the siRNA transfection reagent (Santa Cruz Biotechnology Inc.) according to the manufacturer’s instructions. After 24 h of incubation, an equal volume of fibroblast growth medium 2 was added. The transfection efficiency was evaluated via western blot analysis with antibodies specific for p-Smad 2 and p-Smad 3.

### 4.7. Rat Tail Type I Collagen Gel Contraction Assay

Rat tail type-I collagen was diluted with fibroblast basal medium (CC-3131; Lonza Group, Ltd., Basel, Switzerland) to a concentration of 1 mg/mL and mixed with NPDFs to reach a final concentration of 1 × 10^5^ cells/mL. Following the addition of 1 N NaOH as per the manufacturer’s instructions, 500 μL of the cell-collagen mixture was added into each well of a 24-well cell culture plate. The plate was incubated at 37 °C for 30 min. The cells were then incubated in fibroblast growth medium 2 (cat. no. C-23020, PromoCell, Heidelberg, Germany) overnight and were treated with Fx and TGF-β1. The gel sizes were measured using the ImageJ software (version 1.51j8; National Institute of Health, Bethesda, MD, USA).

### 4.8. Statistical Analysis

Data are presented as mean ± standard error of mean. All statistical analyses were performed using the GraphPad Prism software 5.0 (GraphPad Software Inc., La Jolla, CA, USA). Comparisons between groups were performed by Dunnett’s multiple range tests. Values of *p* < 0.05 were considered to be statistically significant.
